# Primary carcinoid tumor of the gallbladder: A case report and brief review of the literature

**DOI:** 10.1186/1477-7819-8-12

**Published:** 2010-02-23

**Authors:** Yi-Ping Zou, Wei-Min Li, Hao-Run Liu, Ning Li

**Affiliations:** 1 Department of Hepatobiliary Surgery, The PLA 309 Hospital, Beijing, PR China; 2 Pathology, The PLA 309 Hospital, Beijing, PR China

## Abstract

**Background:**

Primary carcinoid tumor of the gallbladder is rare and comprises less than 1% of all carcinoid tumors. Preoperative diagnosis of carcinoid tumor of the gallbladder is difficult. The imageology findings are similar to those in other gallbladder cancers.

**Case presentation:**

A 46-year-old woman was hospitalized with a preoperative diagnosis of gallbladder carcinoma, The patient was referred for surgical opinion and laparotomy was subsequently performed. A 4 × 5 cm mass was found within the gallbladder, located on the free surface of the body and fundus of the gallbladder. Neither metastases nor direct invasion to the liver was found. The entire mass and gallbladder were excised and intact. Histologically, the tumor consisted of small oval cells with round-to-oval neclei and tumor cells formed small nodular, trabeculare and acinar structures. The tumor showed moderate pleomorphism with scattered mitotic figures, but no definite evidence of vascular permeation, perineural invasion or lymphatic permeation was seen. The tumor cells invaded the mucosa extensively, and some penetrated the muscular layer but not through the serosa of the gallbladder into the liver. Immunohistochemical studies revealed strong positive reaction for chromogranin A and NSE. This lesion was proved to be a primary carcinoid tumor of the gallbladder. A brief review of literature, clinical feature, pathology and treatment of this rare disease was discussed.

**Conclusion:**

Primary carcinoid tumor of the gallbladder is uncommon. The definite diagnosis is often made on histopathological results after surgery.

## Background

Generally, carcinoid tumors are thought to arise from embryonal neural crest cells and may occur anywhere that these cells are found. For the most part they tend to be associated with the gastrointestinal tract and respiratory system; however, primary carcinoid tumors of the gallbladder are rare and comprises less than 1% of all carcinoid tumors. We herein present a classical carcinoid tumor found in gallbladder of a 46-year-old woman and review the relevant literature on this rare entity.

## Case Presentation

A 46-year-old woman was hospitalized with a 2-year history of dull pain in the right upper abdomen. Her appetite was normal and she had no history of diarrhea, flushes or dyspnea. There was no pertinent past medical or surgical history. On examination, she was well nourished with stable vital signs, and no pallor, jaundice, or significant lymphadenopathy. Abdominal examination revealed no tenderness, organomegaly, or abnormal mass.

**Figure 1:**
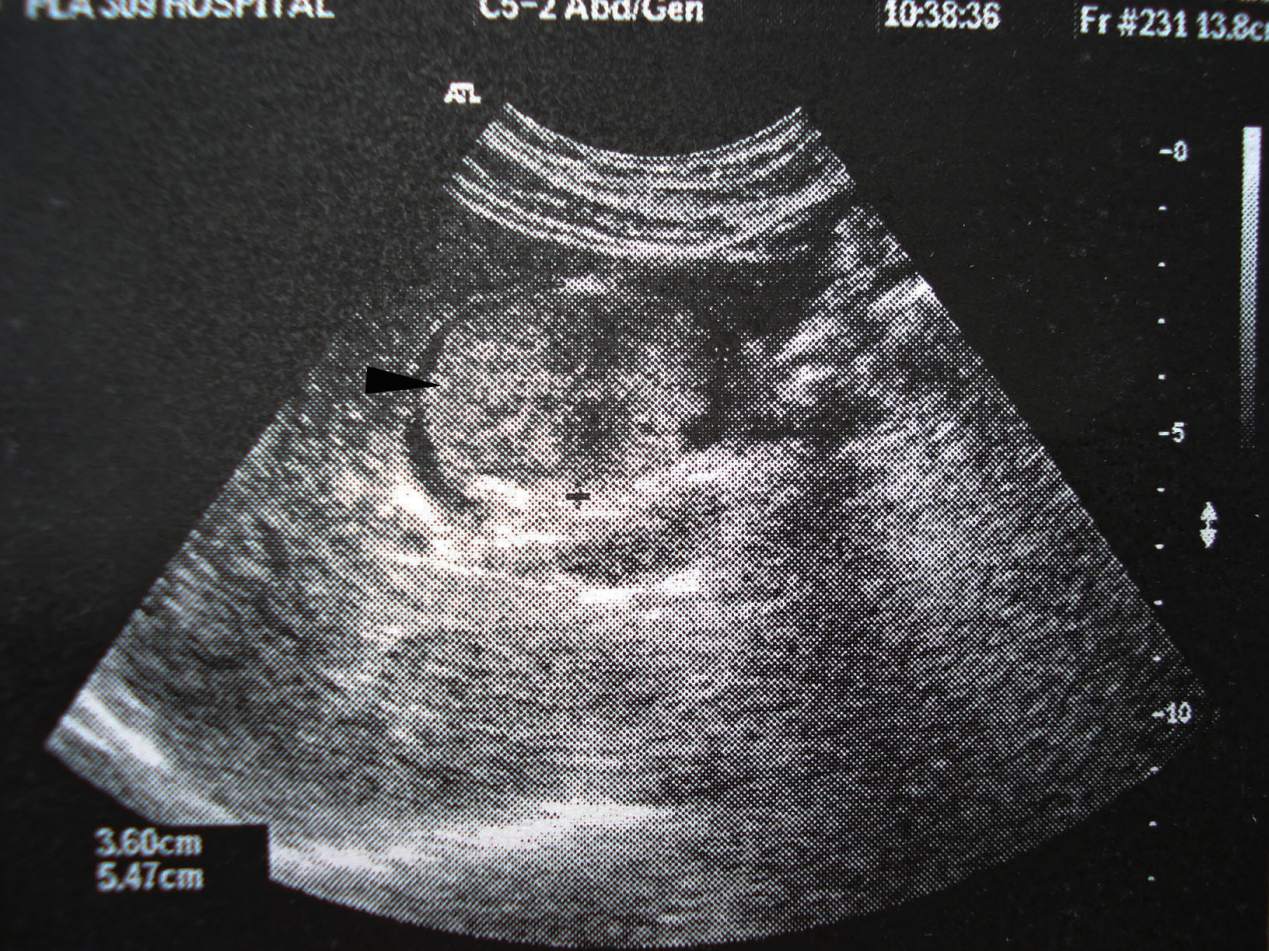
**Abdominal ultrasound examination showing a mass (arrow) in the gallbladder**.

**Figure 2:**
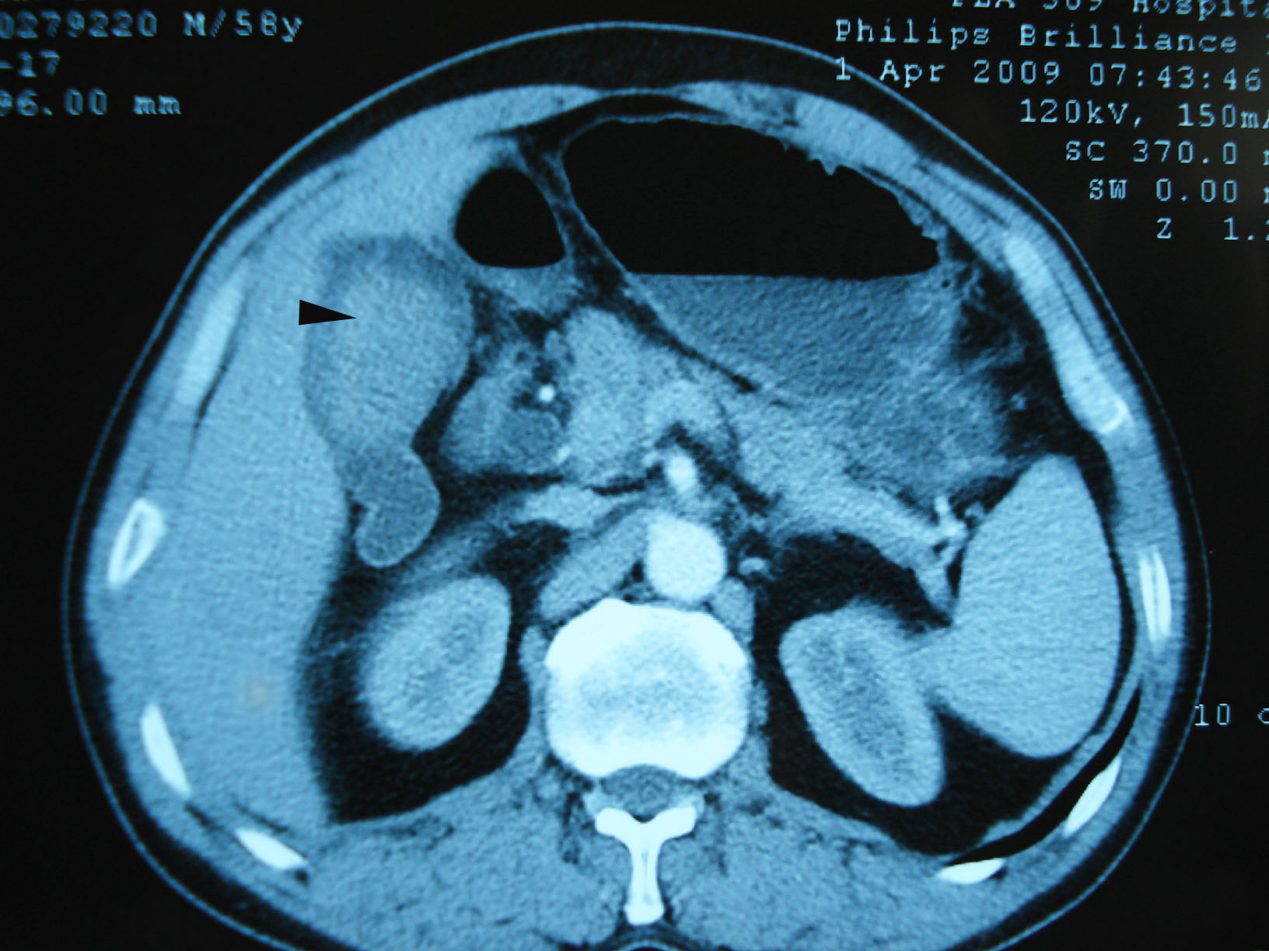
**An abdominal CT scan showing a mass (arrow) at the lumen of gallbladder**.

Laboratory investigation revealed normal hematological findings and serum electrolyte levels. The laboratory data of Liver function were within normal limits. The results of assays for tumor-associated antigen revealed that the serum levels of CEA, CA-50, CA19-9 and CA125 were within normal limits. Urine and stool routine examinations proved normal. Because of no suspicion for the diagnosis of carcinoid tumor before treatment, we did not measure the levels of the urinary 5-hydroxyindoleacetic acid (5HIAA) and plasma serotonin. The chest X-ray revealed no unusual findings. Abdominal ultrasound showed a 4.5 cm protruding tissue mass in the body and fundus of the gallbladder lumen (Fig. 1). This mass appeared to arise from the wall of the gallbladder. Contrast-enhance abdominal computed tomography was performed and revealed a high-density mass in the gallbladder on the atrial phase (Fig. 2). Low-density lesions in the right hepatic lobe were not detected. No evidences of calcification in the mass and biliary dilatation were noted.

**Figure 3:**
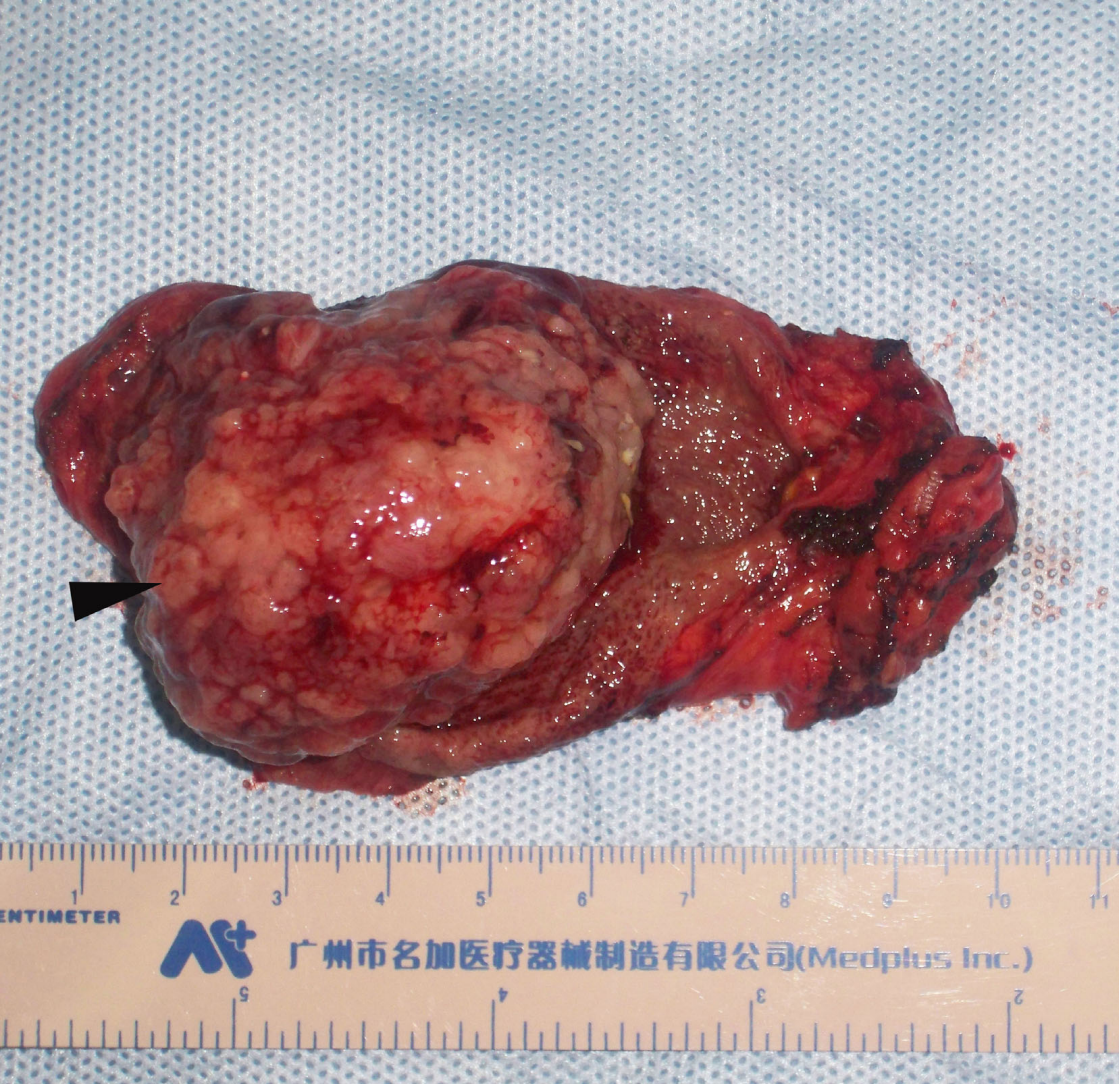
**Resected specimen of the gallbladder presenting a tumor (arrow) in the body and fundus of the gallbladder**.

**Figure 4:**
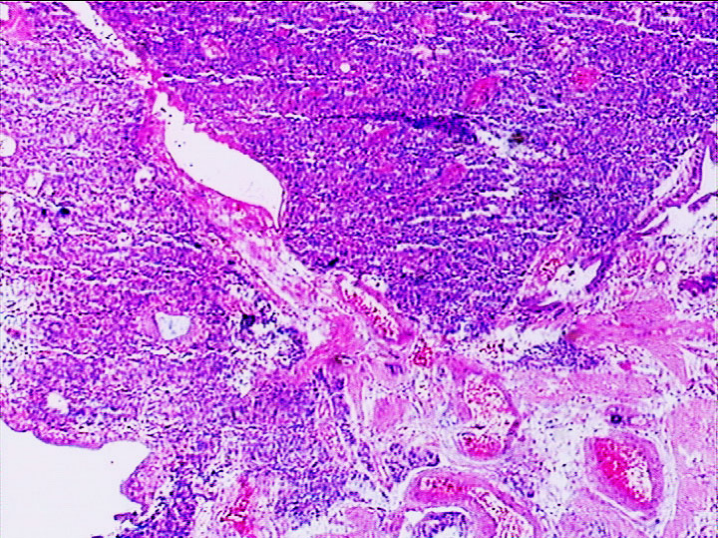
**Hematoxylin & eosin staining showing the tumor cells invaded the mucosa extensively and partially penetrated the muscular layer (original magnification × 4)**.

**Figure 5:**
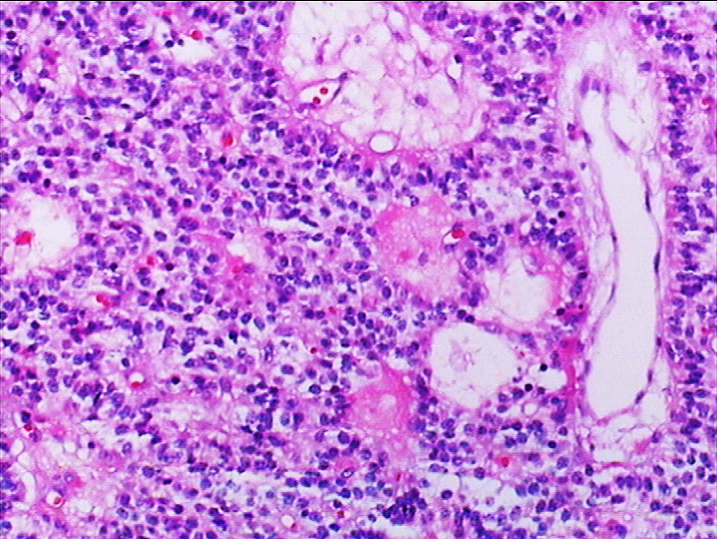
**Hematoxylin & eosin staining showing the tumor consisted of nests of small oval cells with round-to-oval neclei**. Plenty of vascular channels seen between the tumor cells (original magnification × 20).

**Figure 6:**
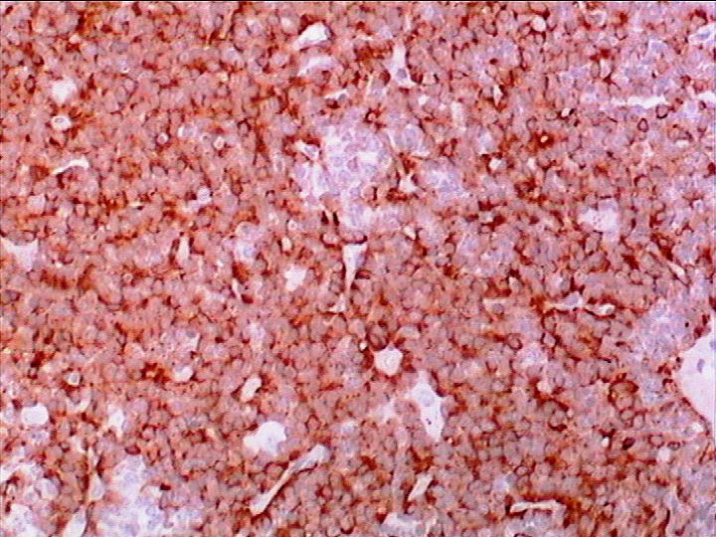
**The tumor cells were diffusely positive for chromogranin A stain (Chromogranin A stain, ×40)**.

**Figure 7:**
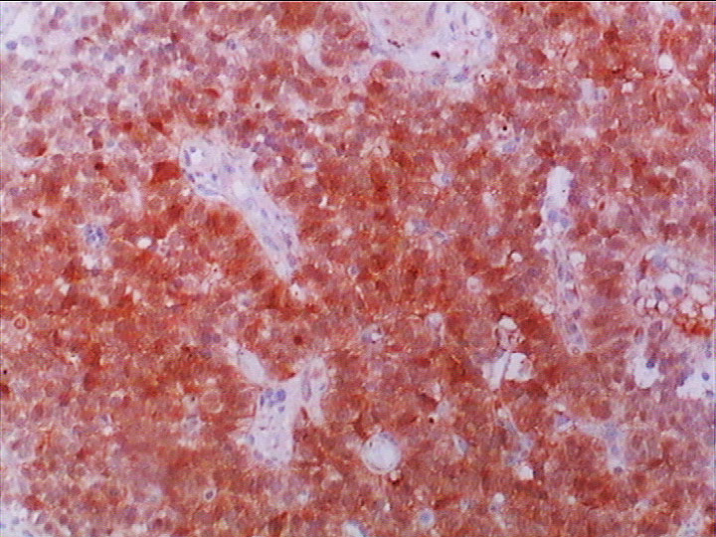
**Neuron-specific enolase staining was positive in most of the tumor cells (NSE stain, ×40)**.

With a preoperative diagnosis of gallbladder carcinoma, the patient was referred for surgical opinion and laparotomy was subsequently performed. At laparotomy, a 4 × 5 cm mass was found within the gallbladder, located on the free surface of the body and fundus of the gallbladder. Neither metastases nor direct invasion to the liver was found. The entire mass and gallbladder were excised and intact. Pathological findings were as follows: On grass inspection of the operated material, the gallbladder measured 10 × 6 × 5 cm, and had a smooth external surface. On opening the specimen, an intramural tumor 5 cm in diameter located in the free wall of the body and fundus of the gallbladder (Fig. 3). Histologically, the tumor was seen infiltrating into the mucosa extensively, and some penetrated the muscular layer but not through the serosa of the gallbladder into the liver. The gallbladder with tumor was completely excised with free resection margins. The tumor consisted of nests of small oval cells with round-to-oval neclei and these nests were separated from each other by thin fibrovascular bands. The tumor showed moderate pleomorphism with scattered mitotic figures, but no definite evidence of vascular permeation, perineural invasion or lymphatic permeation was seen (Fig. 4 and Fig. 5). Immunohistochemical studies of paraffin sections revealed strong positivity for chromogranin A (Fig. 6) and neuron-specific enolase (NSE) (Fig. 7). It was diagnosed as a classical carcinoid tumor of the gallbladder. After surgery, the patient had an uneventful recovery without incident. No recurrent lesion was found using abdominal ultrasound examination and CT scan 12 months after cholecystectomy.

## Discussion

Carcinoid tumors are relatively rare endocrine tumors arising principally in the gastrointestinal tract, where it comprises less than 2% of all primary gastrointestinal tumors [1]. Primary carcinoid tumors are mostly found in the appendix, jejunum and rectum. Less common sites include the bronchial epithelium, duodenum, colon and stomach. The gallbladder in particular is extremely rare site for carcinoid. Sanders [2] reported only 7 tumors (0.2%) in the gallbladder among 3633 digestive tract carcinoids. Godwin [3] also reported only one case (0.04%) in the gallbladder among 2837 carcinoids. The first primary carcinoid tumor of the gallbladder was described by Joel in 1929 [4], and in our investigation to date, only 47 cases of carcinoid tumor of the gallbladder, including that of our patient, were reported in the world English literature [5-14]. From published data including our case, the age of patients ranged from 38 to 81 years [12]. The sex distribution of these lesions paralleled that of gallbladder carcinomas, with a marked female predominance that accounts for 75% of cases in the largest series to date [15]. The most common presentation includes vague abdominal pain or discomfort and associated cholelithiasis [16]. In most instances, they usually lack specific symptoms. Only 3.3%-3.7% of gallbladder carcinoid tumors manifest with carcinoid syndrome [10-16]. Preoperative diagnosis of carcinoid tumor of the gallbladder is difficult. The diagnosis is rarely made by imageology, because most patients are with no specific symptoms and imageology findings are similar to those in other gallbladder cancers. As in the present case, a mass in the gallbladder was indentified but determination of histologic type of tumor and diagnosis to differentiate from gallbladder adenocarcinoma is often difficult. Most carcinoids of the gallbladder were diagnosed incidentally upon routine histological examination of gallbladder specimens at autopsy, after cholecystectomy for cholecystitis, or after surgical treatment of patients in whom a biliary malignancy was suspected [8-16]. Preoperative diagnosis of carcinoid tumor of the gallbladder ordinarily is not possible because of its lack of specific imaging findings.

Mizukami et al [8] and Kaiho et al [9] described in detail the hallmark pathological findings that distinguish the "classical" carcinoid tumors from their "carcinomatous" counterparts. Classical carcinoids of the gallbladder have neither a metastatic nor invasive character and exhibit a more propitious prognosis. The "atypical" variants, however, are associated with marked cell atypia and mitosis, as well as a poor prognosis. From histological analysis, Soga [16] found that 100% of typical carcinoid tumors stain positive for chromogranin A and 93.8% of them stain positive for NSE. In our case, the tumor consisted of small oval cells with round-to-oval neclei and tumor cells formed small nodular, trabeculare and acinar structures. The tumor showed moderate pleomorphism with scattered mitotic figures. The tumor cells invaded the mucosa extensively, and some penetrated the muscular layer but not through the serosa of the gallbladder into the liver. Strong positive reactions for chromogranin A and NSE were observed in almost all tumor cells in the lesion. We think that our case should be diagnosed as a classical carcinoid tumor of the gallbladder.

The majority of reported patients underwent surgery. Surgical strategies have varied from simple cholecystectomy (including laparoscopic cholecystectomy) to extensive hepatic lebectomy, which depended on the size and stage of the lesion, and particularly whether liver metastases were present [5-14]. The SEER database from 1992-1999 indicated that 82.4% of gallbladder carcinoids remain localized and only 11.8% of patients were found with distant metastases [15]. Although some lesions were removed laparoscopically [11], some authors have expressed reservations with regard to laparoscopic excision of gallbladder malignancies since it carries a high risk of port metastases and dissemination [17]. With this consideration, we performed the open cholecystectomy in our case. There is no general agreement on when, or even if, chemotherapy should be started in patients with malignant carcinoid. Conventional chemotherapy including doxorubicin,5-fluorouracil, cisplatin, and streptozocin has minimal efficacy but may have some utility in undifferentiated or highly proliferating neuroendocrine carcinomas. Biotherapy using somatostatin analogs such as octreotide or lanreotide have been assessed in treatment of metastatic disease and remain the only effective pharmacotherapeutic option that improves symptomatology and quality of life with minimal adverse effects [18,19]. The conclusive long-term survival data are limited by the small patient population. Soga [16] collected 138 cases of primary endocrinomas of the gallbladder from the international sources. The results of statistical evaluation showed that the cumulative five-year-survival rate of carcinoid group was 60.4%. From the SEER data (1992-1999), the five-year survival was 58.8 ± 13.3% [15].

Specific prognostic factors have not been identified in patients with gallbladder carcinoids, but increasing tumor size, depth of invasion and metastasis are probably associated with the prognosis [10,16]. Therefore, to improve the prognosis of carcinoid tumor of the gallbladder, it is important to detect the tumor at an early stage and perform curative resection as soon as possible.

Although, the study of neuroendocrine tumors has been advanced significantly by the elucidation of aspects of carcinoid biology and the development of novel diagnostic methodology, there appears to be little change in terms of outcome. The current optimal therapeutic strategy for carcinoid tumors should be based on the appreciation of the obviously malignant yet somewhat restrained biologic behavior of these lesions. It is suggested that the future of the elucidation of this disease process requires correlation with precise cellular and biologic determinants of malignancy as well as delineation of the specific cell of origin and its precise genomic configuration [15]. It will facilitate predictions of the rate of tumor growth and the likelihood of metastatic dissemination, thus allowing optimization of therapeutic intervention.

## Conclusion

Primary carcinoid tumor of the gallbladder is uncommon. It is difficult to differentiate from adenocarcinoma of the gallbladder preoperatively. The definite diagnosis is often made on histopathological results after surgery.

## Consent

Written informed consent was obtained from the patient for publication of this case report and accompanying images. A copy of the written consent is available for review by the Editor-in-Chief of this journal.

## Competing interests

The authors declare that they have no competing interests.

## Authors' contributions

ZYP wrote the initial draft. All authors contributed to the intellectual context and approved the final version. ZYP is the guarantor.
